# Statistical optimization of *Chlorella vulgaris* polysaccharides-mediated synthesis of platinum nanoparticles with dual functions: anticancer and anticoagulant activities

**DOI:** 10.1186/s12934-026-02972-5

**Published:** 2026-03-24

**Authors:** Noura Salah Nour, Sawsan Abd Ellatif, El-sayed Mahdy, Ahmed Atef El-Beih, Hatem El-Mezayen

**Affiliations:** 1 Bioprocess Development Department, Genetic Engineering and Biotechnology Research Institute (GEBRI), City of Scientific Research and Technological Applications (SRTA-City), New Borg El-Arab, Alexandria 21934 Egypt; 2 Biochemistry Division, Chemistry Department, Helwan University, Cairo, Egypt; 3 Chemistry of Natural and Microbial Products Department, National Research Centre, 33 El-Bohouth St., Dokki, Giza, 12622 Egypt

**Keywords:** *Chlorella vulgaris*, Platinum nanoparticles, MCF-7, HepG-2, Plackett-Burman design, Cell factory, Anticancer, Anticoagulant

## Abstract

**Background:**

The biomedical application of platinum nanoparticles (PtNPs) has gained increasing attention in recent years; however, conventional chemical synthesis often depends on harsh conditions and toxic reagents, which restrict their medical use due to concerns of cytotoxicity and environmental hazards. As a result, the development of eco-friendly and biocompatible methods has become a major focus in nanobiotechnology. This study introduces a green method using intracellular polysaccharides from *Chlorella vulgaris* strain ESA251 as reducing and stabilizing agents to create biocompatible CV-PtNPs, evaluating their dual anticancer and anticoagulant activities.

**Results:**

Polysaccharides were extracted from *Chlorella vulgaris* strain ESA251 and characterized by GC-MS, revealing a heteropolysaccharide composition rich in glucose, mannose/galactose, rhamnose, and uronic acid. Platinum nanoparticles were synthesized under optimized conditions (20 mL polysaccharide extract, 40 mL 0.5 mM Hexachloroplatinic acid hexahydrate (H_2_PtCl_6_.6H_2_O), 97 °C, pH 12, 1 h, identified by plackett-Burman design (PBD). UV-Vis, Fourier Transform Infrared spectroscopy (FTIR), Transmission Electron Microscopy (TEM), and Scanning Electron Microscopy (SEM) confirmed the formation of quasi-spherical, well-dispersed nanoparticles predominantly within the nanoscale range (~ 5–20 nm) stabilized by hydroxyl, carbonyl, and ether groups. In vitro assays showed dose-dependent cytotoxicity against Human Breast Adenocarcinoma (MCF-7) and Human Hepatocellular Carcinoma (HepG-2) cancer cells, with IC_50_ values of 20.9 µg/mL and 74.1 µg/mL, respectively. These values suggest promising anticancer activity compared with previously reported chemically synthesized PtNPs. Anticoagulant studies demonstrated inhibition of visible clot formation under the tested in vitro conditions, highlighting them as potential anticoagulant candidates requiring further validation. This dual functionality may provide a basis for future exploration of combined anticancer and anticoagulant strategies, potentially relevant to limiting circulating tumor cell survival.

**Conclusion:**

These findings demonstrate that *Chlorella vulgaris* functions as an efficient microbial platform for the statistically optimized and sustainable biosynthesis of biologically active platinum nanoparticles, supporting their further evaluation in anticancer and anticoagulant research.

## Introduction

Platinum nanoparticles are a key research tool in biomedical science. Their tunable surface plasmon resonance (SPR) is leveraged for applications ranging from detection of pathogens to targeted drug delivery [1]. Designing stable and environmentally benign procedures for producing nanomaterials is a fundamental aspect of nanotechnology [2]. In line with the principle of green chemistry, there is an increasing necessity to avoid or reduce the use of nanomaterials that pose a hazard to the environment [3]. Over the past decade, biological systems have gained attention as eco-friendly alternatives for nanoparticle production [4], using organisms such as fungi, bacteria, algae, and plants, or biomass extracts [5]. These biosynthetic processes are simple, cost-effective, and capable of producing nanostructures with enhanced catalytic activity [6]. Microorganisms, in particular, adapt to environmental stress by producing nanoparticles to detoxify harmful substances and sustain survival, a phenomenon widely reported in literature [7–9].

Among various biotic sources, *C. vulgaris* represents a promising candidate for nanoparticle biosynthesis. This unicellular microalga is known for its high growth rate, strong photosynthetic efficiency, and tolerance to diverse environmental stresses [10]. Known for its high concentration of intracellular polysaccharides (13–19%), *C. vulgaris* produces polysaccharides that rich in sugars like glucose and galactose [11, 12]. These biopolymers are not merely structural compounds but are therapeutically active molecules with wide spectrum of bioactivities.

Microalgal polysaccharides, particularly those from *Chlorella*, are increasingly recognized for their potent biological functions. Their therapeutic potential is largely attributed to their antioxidant, immunomodulatory, and anti-tumor properties [13]. The antioxidnt activity stems from their ability to scavenge free radicals and chelate pro-oxidant metal ions, thereby mitigating oxidative stress [14]. Furthermore, certain polysaccharides can directly inhibit tumor cell proliferation and induce apoptosis via mitochondrial pathways and activation [15]. Importantly, many bioactive polysaccharides exhibit a high degree of biocompatibity and selective toxicity towards malignant cells, making them ideal candidates for developing safer chemotherapeutic adjuvants [16].

The bioactivities of polysaccharide-mediated nanoparticles represent a synergistic combination of the inherent therapeutic properties of the polysaccharide biopolymer and the unique biological effects of the metallic core. This synergy often results in enhanced and multifaceted bioactivity [17]. The polysaccharide shell, rich in functional groups, retains its intrinsic free radical scavenging ability, which is often amplified when conjugated with a metal nanoparticle. Furthermore, the anticancer potential is potentiated through multi-mechanistic processes. The nanoparticles can facilitate selective uptake into cancer cells and, once internalized, disrupt mitochondrial function, generate reactive oxygen species (ROS), and trigger the caspase cascade, leading to programmed cell death (apoptosis) [18]. The polysaccharide coating can also provide a biocompatible platform that enhances colloidal stability and mitigates the inherent toxicity of bare metallic nanoparticles, making them more suitable for biological applications [19, 20].

Building on these intrinsic properties, polysaccharides have proven effective as both reducing and stabilizing agents in the green synthesis of various metallic nanoparticles [13]. The hydroxyl, carboxyl, and amine groups within their saccharide chains efficiently reduce metal ions to their zero-valent state, while simultaneously capping the newly formed nanoparticles to prevent aggregation and confer aqueous stability. This results in a biocompatible “green” shell that can enhance the bioactivity of the metallic core [21].

To date, however, no report exists on the green synthesis of PtNPs using *C. vulgaris* intracellular polysaccharides. Furthermore, the biosynthesis of metallic nanoparticles using algae has been reported, many studies lack a systematic optimization of the critical synthesis parameters (e.g., precursor concentration, pH, temperature, reaction time) [22]. This optimization is paramount, as it directly governs the physicochemical characteristics of the NPs, such as size, shape, and surface chemistry, which in turn dictate their biological functionalities [23]. A significant research gap exists in moving beyond mere synthesis to deliberate, optimized fabrication of NPs with predefined and enhanced bio-activities.

Here, we report for the first time a systematically optimized green synthesis route for PtNPs based on intracellular polysaccharides extracted from *C. vulgaris*. The biosynthesized nanoparticles were characterized using spectroscopic and electron microscopy techniques to examine their size, shape, and physicochemical features.

Nanoparticles have also emerged as promising anticancer agents, with several studies reporting selective cytotoxicity of malignant cells [24]. Hepatocellular carcinoma, the sixth most common malignant tumor in the universe, and Breast cancer, accounting for over 20% of all cancer cases, remain major health challenges [25, 26]. Toxicity and low survival rates continue to limit standard cancer treatments [27], creating an urgent need for novel strategies. One promising approach involves colloidal platinum, which previous research has shown can trigger apoptotic death in esophageal squamous cell carcinoma cells [28, 29]. Building on these findings, the current research investigates the anticancer potential of CV-PtNPs that were prepared using the biological process with *C. vulgaris* intracellular polysaccharides against hepatocellular carcinoma (HepG-2) and breast cancer (MCF-7) cell lines.

## Materials and methods

### Chemicals and test strains

The reagents, including Hexa-chloroplatinic acid (H_2_PtCl_6_.6H_2_O), Sodium Hydroxide, NaNO_3_, K_2_HPO_4_, MgSO_4_.7H_2_O, CaCl_2_.2H_2_O, Citric acid, NaCO_3_, H_3_BO_3_, MnCl_2_.4H_2_O, ZnSO_4_.7H_2_O, Na_2_MoO_4_.2H_2_O, CuSO_4_.5H_2_O, Co(NO)_3_.6H_2_O, Na_2_EDTA, were ordered from Sigma-Aldrich, St. Louis, MO, USA and were of analytical grade. *Chlorella vulgaris* strain ESA251 with accession number (PX603247) was acquired from SRTA-City, Egypt. The human liver carcinoma (HepG-2) and the human breast adenocarcinoma (MCF-7) were acquired from Riken Bioresource Center of Japan (Ibaraki, Japan).

### Growth of algae and preparation of algal polysaccharides extract

For the isolation of pure culture of *Chlorella vulgaris* strain ESA251 algae, a first growth cycle was carried out using BG11 medium [30]. Algal isolates were cultured in BG11 media for 12 days under controlled temperature of 25 °C and shaking conditions at 120 rpm. The resulting biomass was then harvested by centrifugation at 4000 rpm for 20 min., dehydrated at 45 °C, and ground into a powder using a mortar. About 0.5 g of this dried algal extract was then homogenized with 50 mL double-distilled water to extract the polysaccharides using hot water extraction technique [31]. The process involved suspending the powder in deionized water and agitation for 4 h in water bath at 80 °C, centrifugation at 4000 rpm for 20 min. Supernatant thus attained were collected and precipitated overnight at 4 °C with 95% ethanol in 1:4 v/v ratio, and centrifuged at 4000 rpm for 20 min. The precipitate formed was redissolved in deionized water. The supernatant formed was freeze-dried and stored at -20 °C for future use [14].

### Screening of bioactive compounds in the crude intracellular polysaccharides extract of *C. vulgaris*

The bioactive compounds in the algal polysaccharides extract, which were used for green synthesis, were characterized and analyzed using Gas Chromatography-Mass Spectroscopy (GC-MS). The analysis was performed with a specific Shimadzu GC-MS-QP 2010 plus system and a RTX-5 capillary column(60 m × 0.25 mm, film thickness 0.25 μm), using helium as the carrier gas, following an established procedure by Soliman et al. [32].

### Statistical optimization of platinum nanoparticles production

Placket-Burman design was utilized in platinum nanoparticles biosynthesis for screening of six variables. The variables were screened at 2 levels and comprised 9 experimental runs. Placket-Burman design is derived on the basis of the 1st order model:

1 $$Y=\beta0+\sum\beta i+Xi$$

Where Y represents the response variable we seek to predict β0 is the model intercept, βi is the linear coefficient and Xi is the independent variable level. All experiments will run twice, and the average of the results was used as the response. Table 1 outlines factors of investigation and associated levels in the experimental design, both at the symbol level and the actual variable level. These independent variables were seen in 9 combinations, as reported through the symbol code, and the realized levels of these variables are presented in Table 2. The nanoparticles’ concentration was tested with the help of spectrophotometry.

### Synthesis of platinum nanoparticles

Platinum nanoparticles were synthesized by adding 40 mL of a 0.5 mM hexachloroplatinic acid (H₂PtCl₆.6H_2_O) solution to 20 mL of a polysaccharide extract from *C. vulgaris*. The mixture was heated at 97 °C for one hour in the dark. The resulting nanoparticles were then dried in an oven for later characterization [33].

**Table 1:** Nine-trial Plackett-Burman experimental design for evaluation of six independent variables along with experimental & expected SPR of produced CV-PtNPs

Trial	X1	X2	X3	D1	X4	X5	X6	Response	Predicted *R*
1	1	-1	-1	1	-1	1	1	1.391	1.391078942
2	1	1	-1	-1	1	-1	1	1.0767	1.076717037
3	1	1	1	-1	-1	1	-1	2.1032	2.097952163
4	-1	1	1	1	-1	-1	1	0.7704	0.775836031
5	1	-1	1	1	1	-1	-1	1.9323	1.927003957
6	-1	1	-1	1	1	1	-1	1.0066	1.006618163
7	-1	-1	1	-1	1	1	1	1.4221	1.416807497
8	-1	-1	-1	-1	-1	-1	-1	0.5832	0.583282059
9	1	-1	1	-1	1	1	-1	2.3222	2.332963793

The “−1” sign corresponds to the minimum value and the “+1” sign corresponds to the maximum value of the input parameter range, values of response express as mean ± SD (*n* = 3)

**Table 2:** The true values of the coded variables of Plackett-Burman design

Code	Variable	Levels	
		+ 1	-1
X1	Pt salt volume (µ)	400	200
X2	shaking	yes	no
X3	Algal polysaccharide volume(µ)	400	200
X4	pH	11	9
X5	Temperature (^o^C)	90	70
X6	Time(h)	2	1

### Characterization of Pt nanoparticles

#### UV-Vis analysis

The biological synthesis of CV-PtNPs was qualitatively confirmed visually through visible color change observation. UV-absorption Spectra of CV-PtNPs biosynthesized were quantitated by performing Ultraviolet-visible spectroscopy UV-Vis on a Shimadzu UV-2700 spectrophotometer. Biosynthesized PtNPs between 200 and 600 nm were scanned at the same intervals to track Pt^+ 4^ reduction to PtNPs while a polysaccharides solution served as control. All UV-Vis spectral graphs were processed and plotted using R statistical software to ensure high graphical quality, uniform formatting and reproducibility.

#### FTIR analysis

A sample biosynthesized PtNPs Spectra was obtained by making a pellet with KBr in the ratio 1:100. The FTIR analysis was done by Shimadzu FTIR-8400s equipment in the range of (4000 and 400 cm^− 1^) spectral resolution with 1 cm^− 1^. This analysis was performed to verify whether functional groups represent in *C. vulgaris* polysaccharides extract are responsible for reduction and stabilization. FTIR spectra were replotted using R software to improve visualization quality and consistency of graphical presentation.

#### SEM analysis

For more morphological examinations of biogenically prepared Pt nanoparticles, Scanning Electron Microscopy (SEM) was employed using a Hitachi S-4500 SEM.

#### TEM and energy-dispersive X-ray spectroscopy

Transmission electron microscopy (TEM) measurements were done by the method of ref. [34] on JEOL GEM-1010 Transmission electron microscope at 15 kV [35]. The chemical composition of the synthesized platinum nanoparticles was determined by energy-dispersive X-ray spectroscopy (EDX) using a JEM-2100 F field-emission transmission electron microscope (JEOL Ltd., Tokyo, Japan) operated at an accelerating voltage of 200 kV. The microscope was equipped with an EDAX Octane T Ultra W silicon drift detector (SDD) with a super-ultra-thin window (SUTW).

#### Particle size analysis

The particle-size distribution of platinum nanoparticles powder was determined using a laser diffraction analyzer (Mastersizer S, Malvern Instruments, UK). This analysis was conducted at the Central Laboratory of the Advanced Technology and New Materials Research Institute in New Borg El-Arab City, Alexandria, Egypt.

### Anticoagulant activity of CV-PtNPs

In order to check the anticoagulant activity of CV-PtNPs, the method developed by ref. [36] was used. Briefly, the anticoagulant activity of CV-PtNPs was investigated by mixing blood freely donated by a healthy volunteer with equal volume of 800 µg/mL of nanoparticles. The control samples were setup using EDTA and H_2_PtCl_6_.6H_2_O solution. The reaction mixtures were held at room temperature (30 ± 2 °C) for 1 h and the anticoagulant activity of the CV-PtNPs was examined.

### Determination of cytotoxicity

The biosynthesized CV-PtNPs were screened against two cancer cell lines; Human Breast Adenocarcinoma (MCF-7) and Human Hepatocellular Carcinoma (HepG-2). The MTT assay was used to find out the cytotoxicity of these Pt nanoparticles.

The cell lines and culturing cells were grown in Dulbecco’s modified eagles medium (DMEM, Wako Pure Chemical Industries, Osaka, Japan) supplemented with 10% fetal bovine serum (FBS) and 100 U/mL penicillin-streptomycin at 37 ℃ in a humidified 5% CO_2_ incubator. Cells were routinely tested negative for mycoplasma. The cells were treated with Pt-nanoparticles sample after 24 h. 5-fluorouracil was the positive control.

### MTT (3-(4,5-dimethylthiazol-2-yl)−2,5-diphenyltetrazolium bromide) assay

The cytotoxicity of platinum nanoparticles were tested against MCF-7 and HepG-2 using MTT colorimetric assay according to the method of [37]. The percentage of cytotoxicity was calculated by the next equation:

2 $${{\% ~Cytotoxicity = }}\left[ {{\mathrm{1}} - \frac{{{\mathrm{Mean~absorbance~of~treated~wells~(X)}}}}{{{\mathrm{Mean~absorbance~of~negative~control~(NC)}}}}} \right] \times {\mathrm{100}} $$

Where X represent the average absorbance of the sample wells at 570 nm, and NC corresponds to the average absorbance of the negative control wells at the same wavelength [38]. IC_50_ was determined by regression analysis of the dose response curve using GraphPad Prism 10.

### Statistical analysis

The statistical analysis of the experimental data was performed using the Microsoft Excel^®^ software package. Results are presented as the mean ± standard deviation derived (SD) from replicate measurements. To determine the significance of observed differences between experimental groups, a one-way analysis of variance (ANOVA) was employed. A probability value (p-value) of less than 0.05 was established as the threshold for statistical significance.

## Results and discussion

This work aimed at the green synthesis and characterization of platinum nanoparticles (CV-PtNPs) utilizing *C. vulgaris* intracellular polysaccharides, followed by evaluation of their anticoagulant and cytotoxic effects against MCF-7 and HepG-2.

The key parameters influencing nanoparticle synthesis were identified and screened using a Plackett-Burman design (PBD), which was subsequently applied for the preparation of PtNPs.

### Statistical optimization of platinum nanoparticles production

Plackett-Burman design (PBD) was used in screening of 9 different runs of 6 independent variables. Experimental design of Plackett-Burman design for 9 runs is demonstrated in Table 1, and the response for platinum nanoparticles production based on SPR at 215 nm is shown. CV-PtNPs production showed severe variation across the range of parameters investigated, from 0.582 to 2.322 for the nine runs. The enormous variation points to the requirement for the precise control of the growth medium to obtain increased production. The maximum production was noted in run 9, which utilized minimum shaking and time levels, and with the highest platinic acid, polysaccharides, pH, and temperature levels. Conversely, the lowest production was recorded for run 8 when the 6 variables were at their lowest levels.

**Fig. 1:**
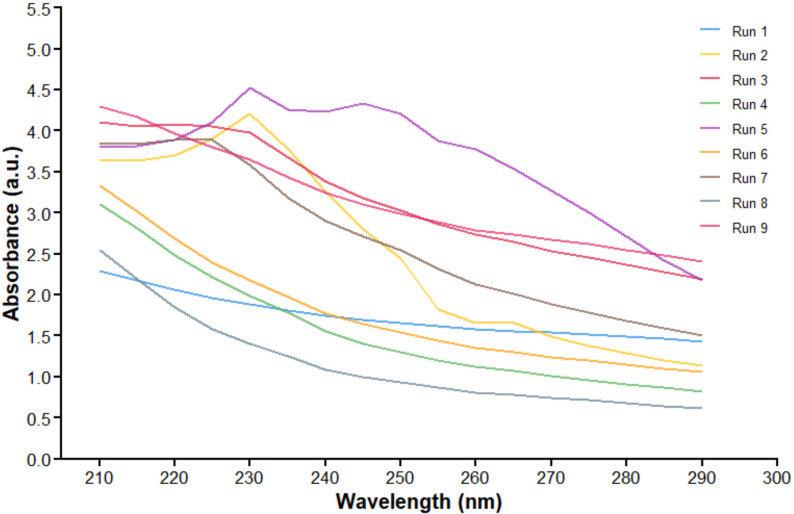
Comparative UV-Vis spectra of optimization experiments showing 9 runs as determined by Plackett-Burman design

**Fig. 2:**
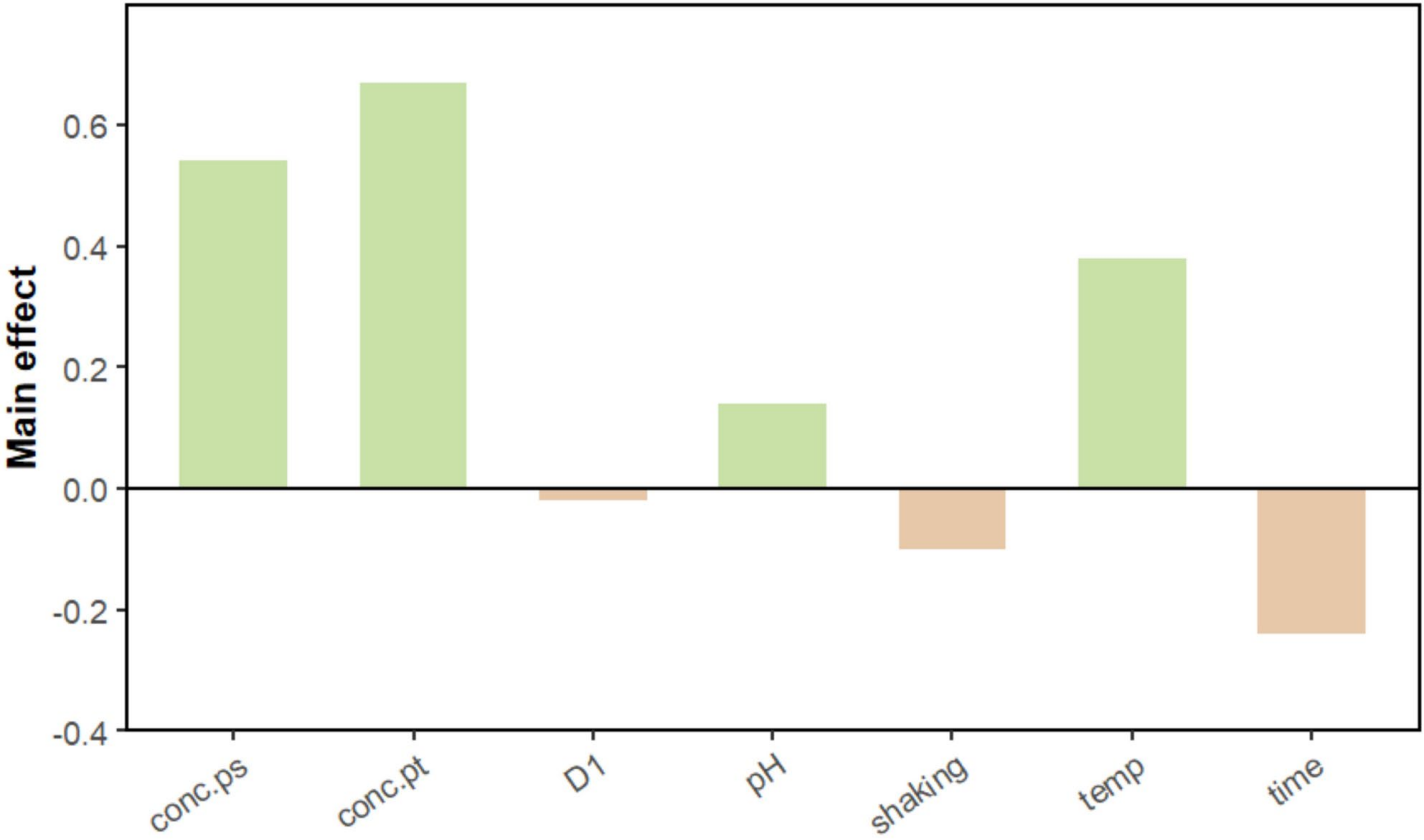
The main effect of variables of Plackett-Burman design for CV-PtNPs

Figure 1 indicates the UV-Vis spectra and color change for the nine runs of the Plackett-Burman design. Figure 2 is the graphical representation of the individual effect of each variable on CV-PtNPs production and illustrates the overall impact of every factor by comparing the average measurements taken at the upper (+ 1) and lower (-1) levels of every factor. ANOVA of the experimental design was carried out by Excel, Microsoft^®^ Office 2021 (Microsoft Corporation, Redmont, WA, USA). The importance of the model was obtained or determined with the help of the p-value, which is used to test the importance of every parameter. The high F-value of 1846 suggested the importance of the model. Model terms with “Prob F” (p-value) below 0.05 are considered important. Table 3 is the statistical data analysis of CV-PtNPs production using the Plackett-Burman design, from which platinic acid concentration, polysaccharides concentration, pH, temperature, and time are recognized as significant model terms. On the other hand, model terms having values greater than 0.1 are not considered important. It’s clear that the three factors, platinic acid concentration, polysaccharides concentration, and temperature, had a positive effect on CV-PtNPs production, which implies that raising the concentration of these factors could increase CV-PtNPs production. On the other hand, shaking and incubation time had a negative effect on PtNPs production. Therefore, the concentration of platinic acid will be chosen for additional optimization (Box-Behnken design), alongside the concentration of polysaccharides and temperature. This is in concordance with Hang, H. et al. Who mixed catkin extract with AgNO_3_ and incubated at varying temperatures and varying concentrations of catkis extract and AgNO_3_ to optimize the biosynthesis of silver nanoparticles [39].

The value of the determination coefficient R^2^ at 0.9999 also means that almost 99.99% of CV-PtNPs production variation is explained by the independent variables of the model, while only around 0.01% of the total variation remains unexplained. Moreover, the adjusted R^2^ value of 00.999 further highlights the strong reliability of the model [40].

**Table 3:** Statistical analysis of key variables using Plackett-Burman Design

	Coefficients	*P*-value	Lower 95%	Upper 95%
Intercept	1.284412	0.002564045	1.218681301	1.350143
Conc.pt ion	0.338776	0.009720441	0.273045363	0.404507
Shaking	-0.04513	0.072654897	-0.110861813	0.0206
Conc.ps	0.269988	0.012196491	0.20425725	0.335719
D1	-0.00928	0.32381567	-0.075008388	0.056453
pH	0.072375	0.045426342	0.006644002	0.138105
Temp.	0.193702	0.016997874	0.12797153	0.259433
Time	-0.1193	0.027587499	-0.185032785	-0.05357

### In-vitro biosynthesis of PtNPs

Platinum nanoparticles (CV-PtNPs) were successfully synthesized by mixing hexachloroplatinic acid with a polysaccharides extract from *C. vulgaris*. The optimal reaction conditions were determined to be: 20 mL of polysaccharides extract at a concentration of 21.65 mg/mL, 40 mL of H_2_PtCl_6_.6H_2_O acid solution with a concentration of 0.5 µM, 97 ℃, pH 12, and a 1 h reaction time.

**Fig. 3:**
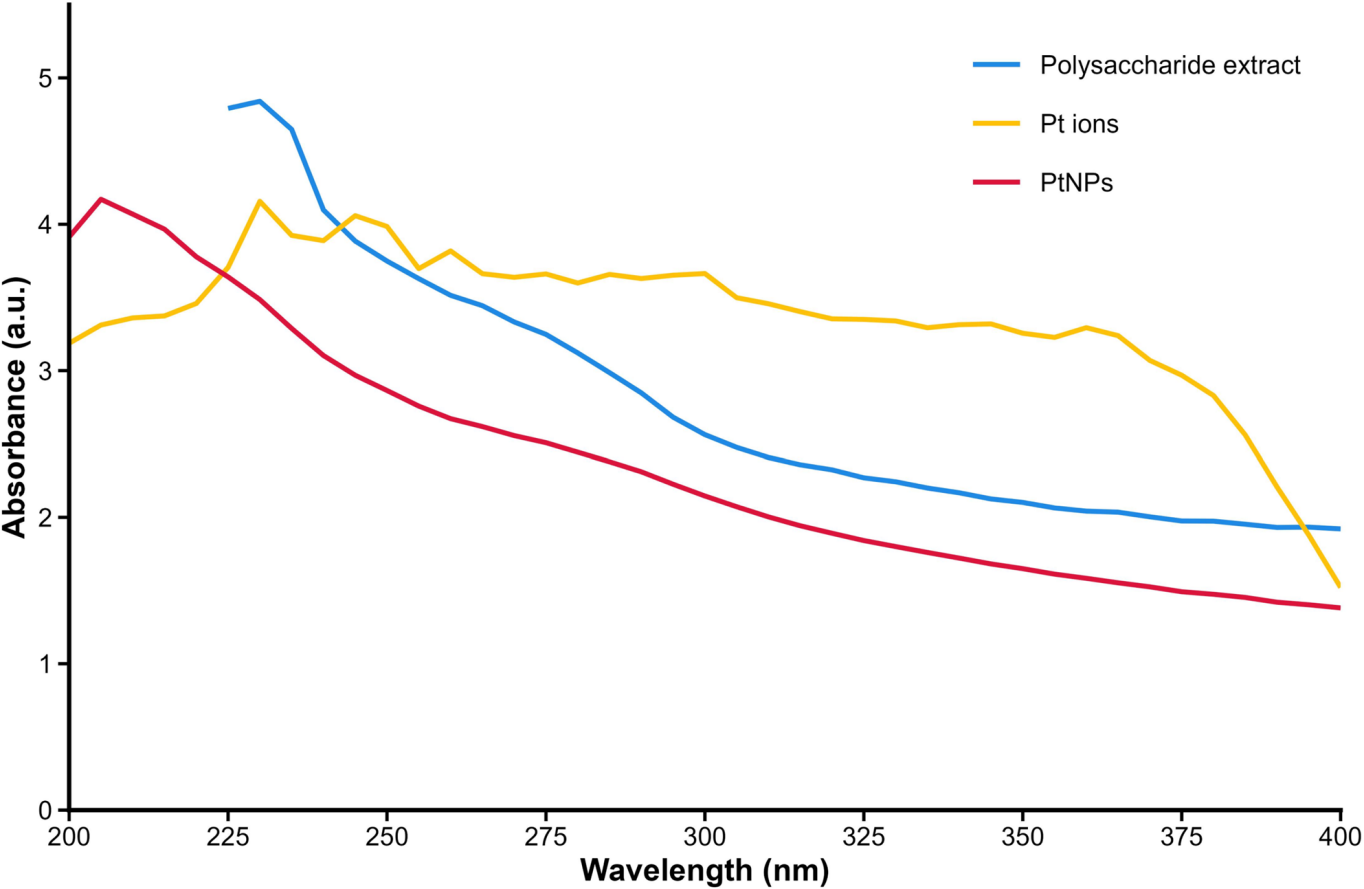
UV-Vis spectra of intracellular polysaccharides extract of *Chlorella vulgaris*, platinum precursor solution (Pt ions), and biosynthesized platinum nanoparticles (CV-PtNPs). The characteristic spectral changes confirm the reduction of Pt ions and formation of nanoparticles

A visible color change from yellow to brown, (Fig. 3**)**, confirmed the nanoparticle formation, which did not occur in control experiments. The polysaccharide extract is essential as it provides the bioactive compounds that act as both reducing and stabilizing agents.

### Physical characterization of optimized CV-PtNPs

#### UV-Vis studies of platinum nanoparticles

UV-Vis spectroscopy, a standard tool for confirming nanoparticle synthesis, was used in this study to verify the formation of biosynthesized platinum nanoparticles. In Fig. 3, we can see the UV-visible Spectra for the platinum nanoparticles biosynthesized by *C. vulgaris* polysaccharides, and with the relevant control. It shows that the UV-Vis absorption spectrum for the polysaccharides lacks any distinct peaks in 260–280 nm region. This indicates that the extract has traces of protein or nucleic acids since these biomolecules are typically marked by absorbance at approximately 280 nm and 260 nm, respectively [41]. For the bio-synthesized platinum nanoparticles sample, the absorbance was taken after stirring the solution magnetically for one hour at 90 ℃. Then metal Chloro-complexes [PtCl_4_]^2-^ in the precursor solution, having characteristic bands in the UV-visible region corresponding to ligand-p to metal-d charge transfer, will not affect the absorption. Therefore, those complexes would exert absorption peaks in the UV-visible spectrum. On the other hand, Pt(0) nanoparticles don’t possess the capability to show ligand-p to metal-d charge transfer, and thus lead to the lack of characteristic bands in the UV-visible spectrum [42]. The absence of characteristic absorption peaks here suggests substantial reduction of Pt ions and nanoparticle formation [43]. The initial absorption peak was lost subsequently [44]. The change of spectrum was a solemn call for formation [45]. The same outcome was observed for by synthesis of platinum nanoparticles from *Psidium guajava* extract [44].

#### Fourier transform infrared spectroscopy (FTIR)

The FTIR spectral analysis of the extracted *C. vulgaris* polysaccharides, (blue line), and the biosynthesized PtNPs, (red line**)**, were analyzed to identify the functional groups involved in nanoparticles formation. The spectrum of the crude polysaccharides showed a broad absorption band at ~ 3400 cm^-1^ corresponding to O-H stretching vibrations of hydroxyl groups, and a peak around 2920 cm^-1^ due to C-H stretching of aliphatic chains. Strong absorption near 1650 cm^-1^ indicated the presence of C = O stretching of carbonyl groups, while peaks in the range of 1000–1150 cm^-1^ were due to C-O-C stretching vibrations of glycosidic linkages [46–50].

**Fig. 4:**
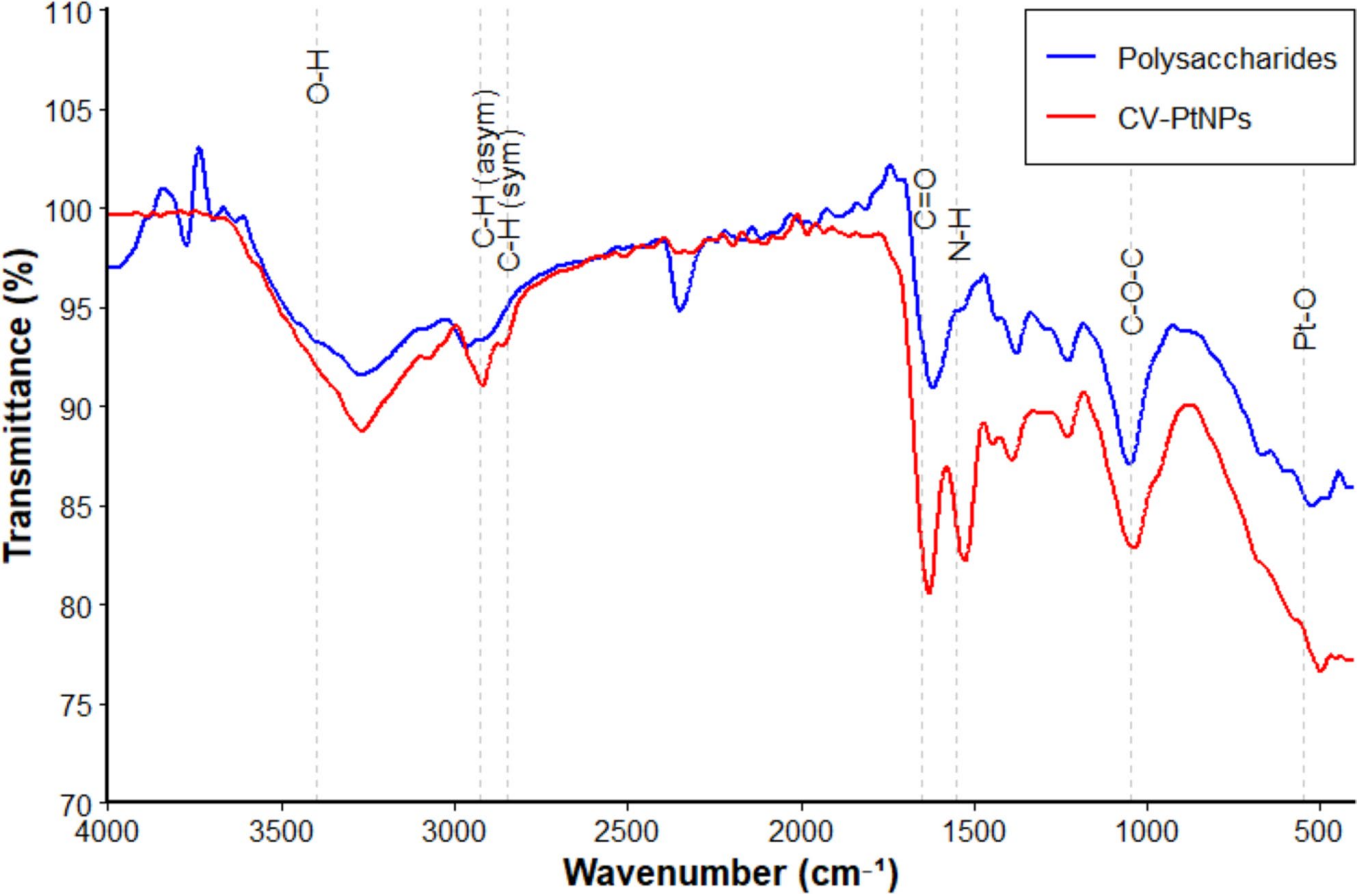
Characterization of CV-PtNPs using FTIR analysis of *C. vulgaris* crude polysaccharides (blue line) and CV-PtNPs (yellow line). Shifts in major functional groups (O–H, C–H, C = O, and C–O–C) indicate involvement of polysaccharide hydroxyl and carbonyl groups in reduction and stabilization of platinum nanoparticles

Upon CV-PtNPs formation, the FTIR spectra (Fig. 4) showed noticeable shifts and intensity reductions were noticed in the O-H and C = O stretching bands, supposing their direct involvement in the reduction of Pt^4+^ ions to Pt^0^. The broadening of the C-O-C absorption region is also reflected coordination of ether linkages with the nanoparticles’ surface, acting as stabilizing or capping agents [51]. These spectral changes confirm that hydroxyl groups acted as electron donors, carbonyl groups had a role in reduction reactions, and ether linkages helped in nanoparticle stabilization, as shown in Table 4. A similar finding was observed in the biosynthesis of Selenium nanoparticles that were stabilized with polysaccharides of *Lactococccus lactis* [52] (Fig. 4).

**Table 4:** FTIR peak assignments of *C. vulgaris* polysaccharides and CV-PtNPs

Wave number cm^− 1^)	Assignment	Polysaccharides (CV-PS)	PtNPs	Interpretation
~ 3400	O-H stretching (hydroxyl groups)	Strong, broad	Shifted and reduced	Hydroxyl groups donate electrons, reduce Pt4+
~ 2930	C-H stretching (aliphatic chains)	Present	Slightly reduced	Minor interaction, structural backbone retained
~ 1650	C = O stretching (carbonyl groups)	Strong	Shifted and weaker	Carbonyl groups involved in reduction of Pt ions
~ 1400	C-H bending/ COO- vibrations	Moderate	Reduced	COO- participates in electrostatic interaction

#### Scanning electron microscope (SEM) analysis

**Fig. 5:**
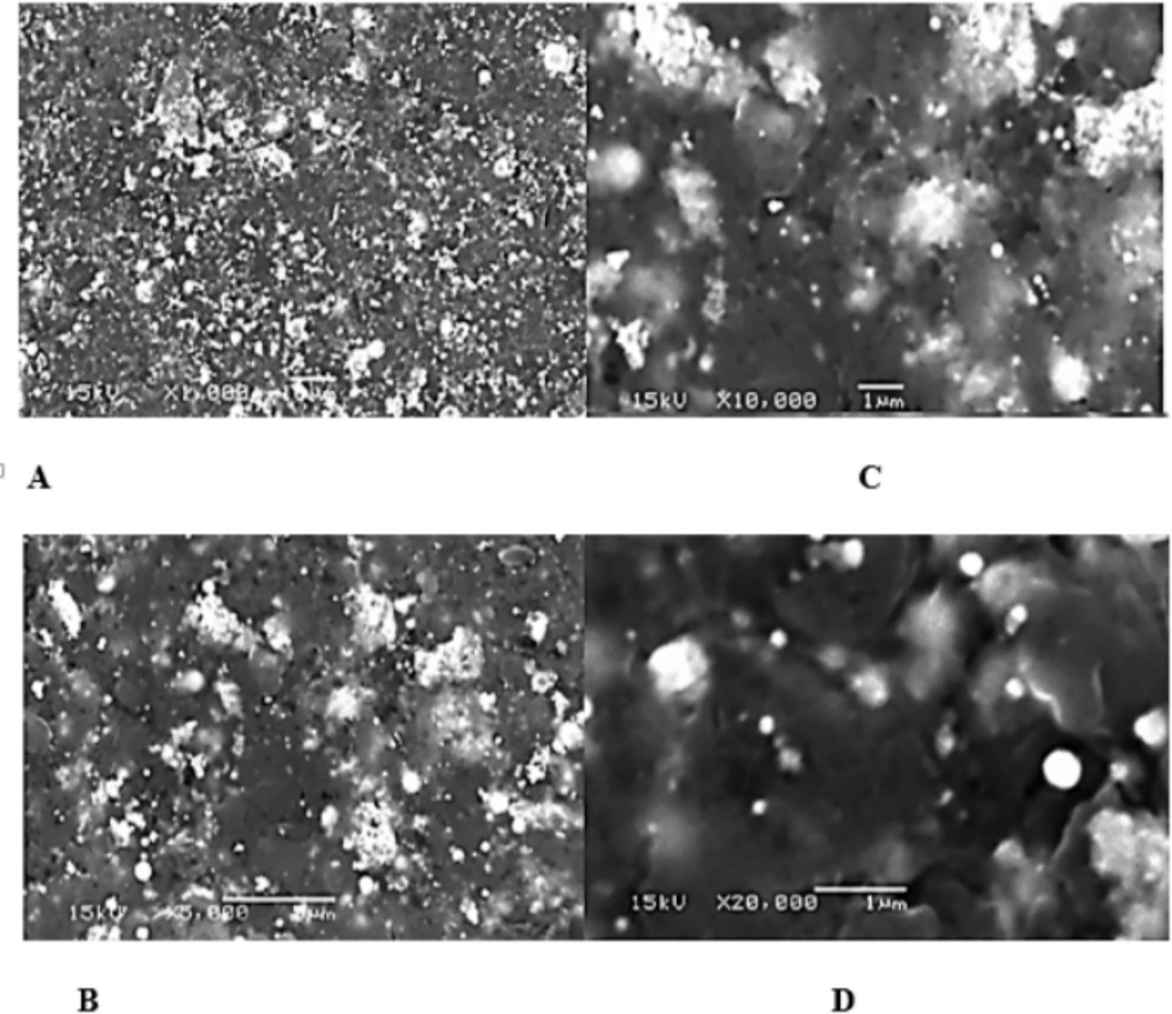
SEM micrographs of bio-synthesized platinum nanoparticles using Chlorella vulgaris (CV-PtNPs) at different magnifications:(A) ×1,000, showing the overall surface distribution of nanoparticles;(B) ×5,000, revealing increased visibility of nanoparticle clusters;(C) ×10,000, showing clearer aggregation and distribution patterns;(D) ×20,000, highlighting densely aggregated nanoparticles with quasi-spherical morphology. *Chlorella vulgaris*

SEM analysis was used to screen for the nanoparticles’ characteristics such as size, shape, and morphological characteristics. Figure 5 shows the SEM image of the CV-PtNPs that were biosynthesized. Different magnifications were utilized to take the SEM image. From SEM data, PtNPs average size ranges between 7 and 20 nm. The CV-PtNPs, shown as white dots in the image, are spherical and dense. These observations were consistent with those of Ali and Mohammed [53].

#### Transmission electron microscope (TEM)

**Fig. 6:**
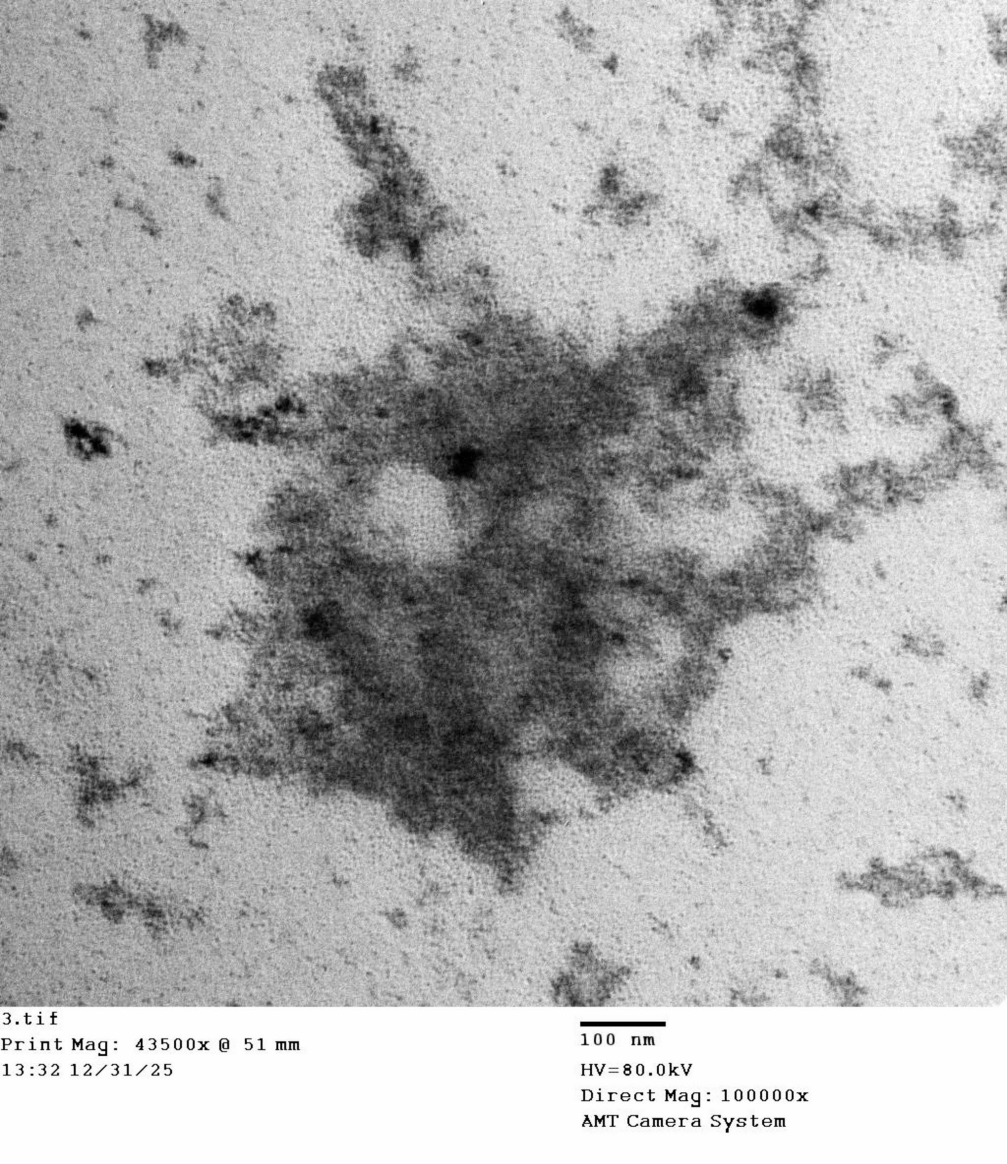
TEM micrograph of bio-synthesized platinum nanoparticles using *Chlorella vulgaris* (CV-PtNPs) showing quasi-spherical nanoparticles with partial aggregation. Scale bar = 100 nm

TEM image, (Fig. 6), shows the metallic nanoparticles as dark area. The sizes are between 5 and 18 nm. The figure shows that the quasi-spherical platinum nanoparticles are present with reduced particle size and low polydispersity, in high yield. The TEM image shows spherical and irregular aggregated particles with sizes in the range of 5 to 18 nm on average. Similar findings were reported in the research of Gamma-Lara, et al. [54].

#### EDX analysis of CV-PtNPs

**Fig. 7:**
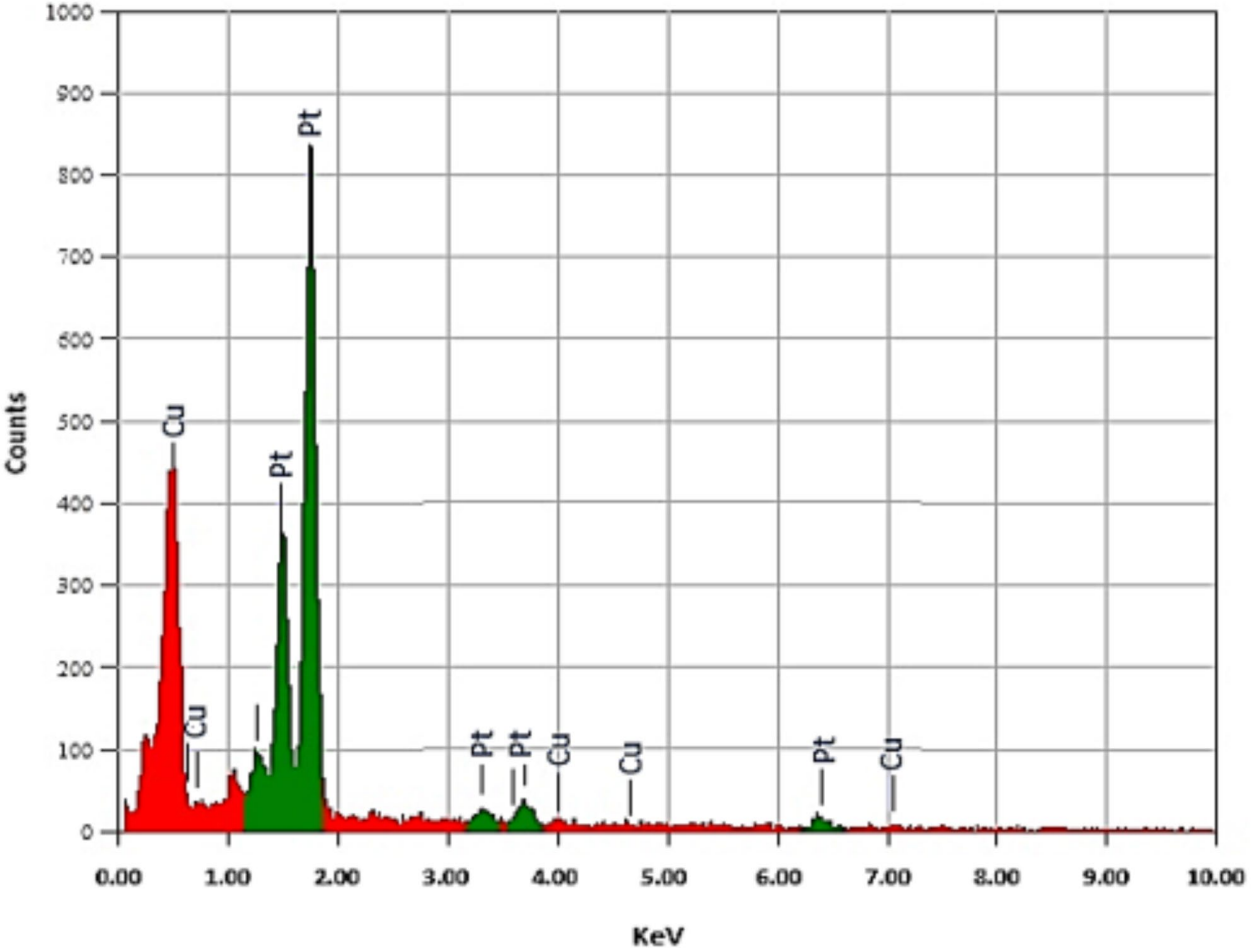
Energy-dispersive X-ray spectroscopy spectra of bio-synthesized platinum nanoparticles using C*hlorella vulgaris* (CV-PtNPs)

Energy-dispersive X-ray spectroscopy (EDX) analysis of bio-synthesized platinum nanoparticles using *chlorella vulgaris* (CV-PtNPs) confirmed the existence of platinum as the dominant element in the nanoparticles (Fig. 7). A representative EDX spectrum (Fig. 7) exhibited intense Pt (1.15–1.86 keV) and Pt (3.2–3.8 keV) peaks.

#### Particle size analysis (PSA)

**Fig. 8:**
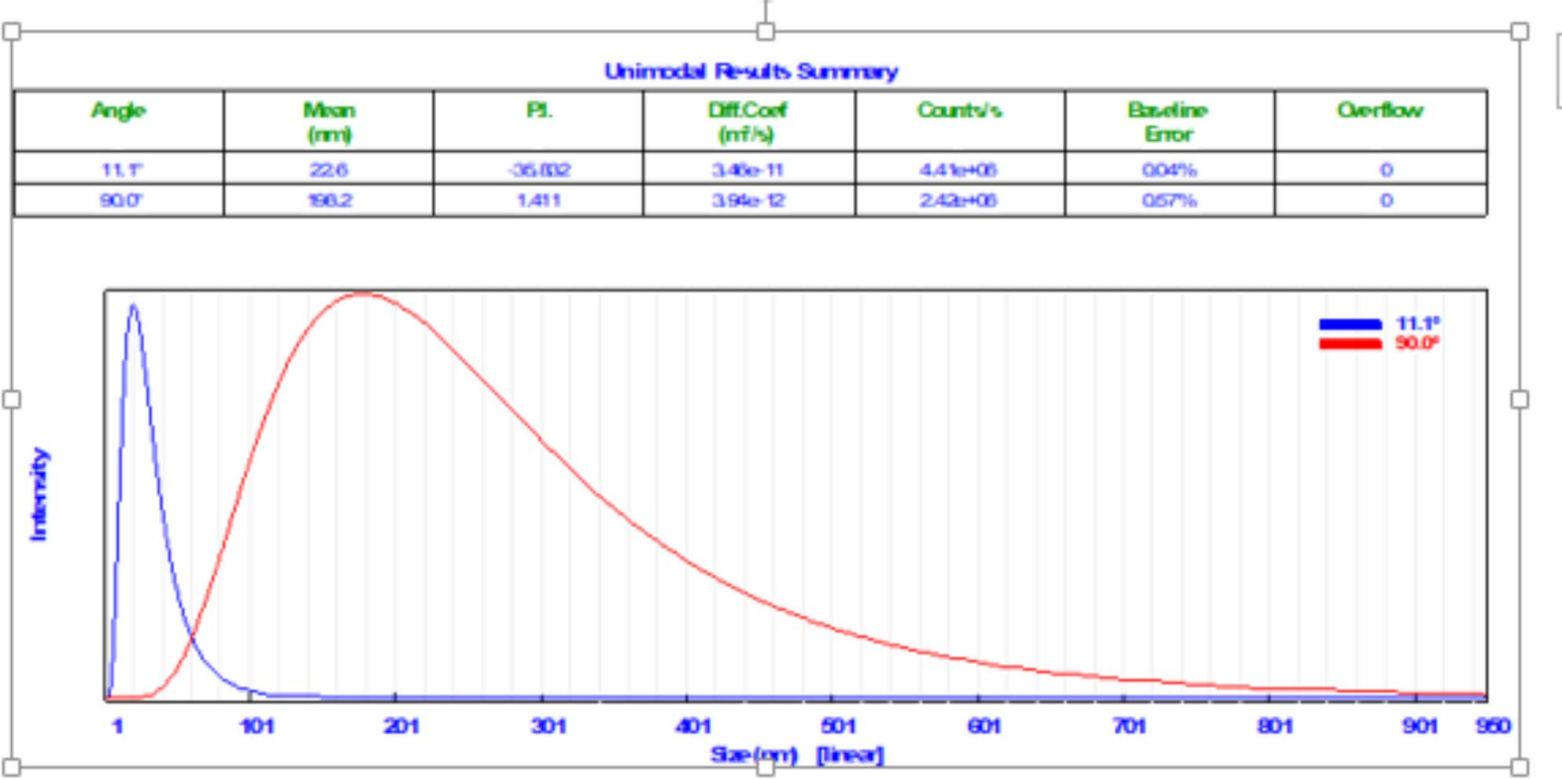
Particle size distribution of biosynthesized PtNPs using *C. vulgaris* polysaccharides as a reducing and stabilizing agent under all optimized conditions

The average particle size was 7–20 nm as shown in Fig. 8. Particles were predominantly within the nanoscale range (5–20 nm), as observed consistently acress TEM, SEM, and PSA analyses. It is important to note that the spectral profile obtained in Fig. 7 differs visually from the earlier spectra because it was generated using the device’s default acquisition mode, which applies an internal baseline and smoothing algorithm that cannot be modified by the user. As a result, the instrument produces a spectrum with slightly different scaling and background characteristics compared to the manually processed spectra shown in Figs. 1, 2, 3, 4, 5 and 6. Despite these differences, the characteristic signals relevant to platinum and the sample’s optical response are still clearly identifiable and remain consistent with the previously presented results.

### Screening of the bioactive compounds presented in algal polysaccharides

To investigate the bioactive compounds present in the polysaccharide extract from *C. vulgaris* and play a role in Pt nanoparticles formation, GC-MS analysis was applied as follows.

### GC-MS analysis of the algal polysaccharides

**Fig. 9:**
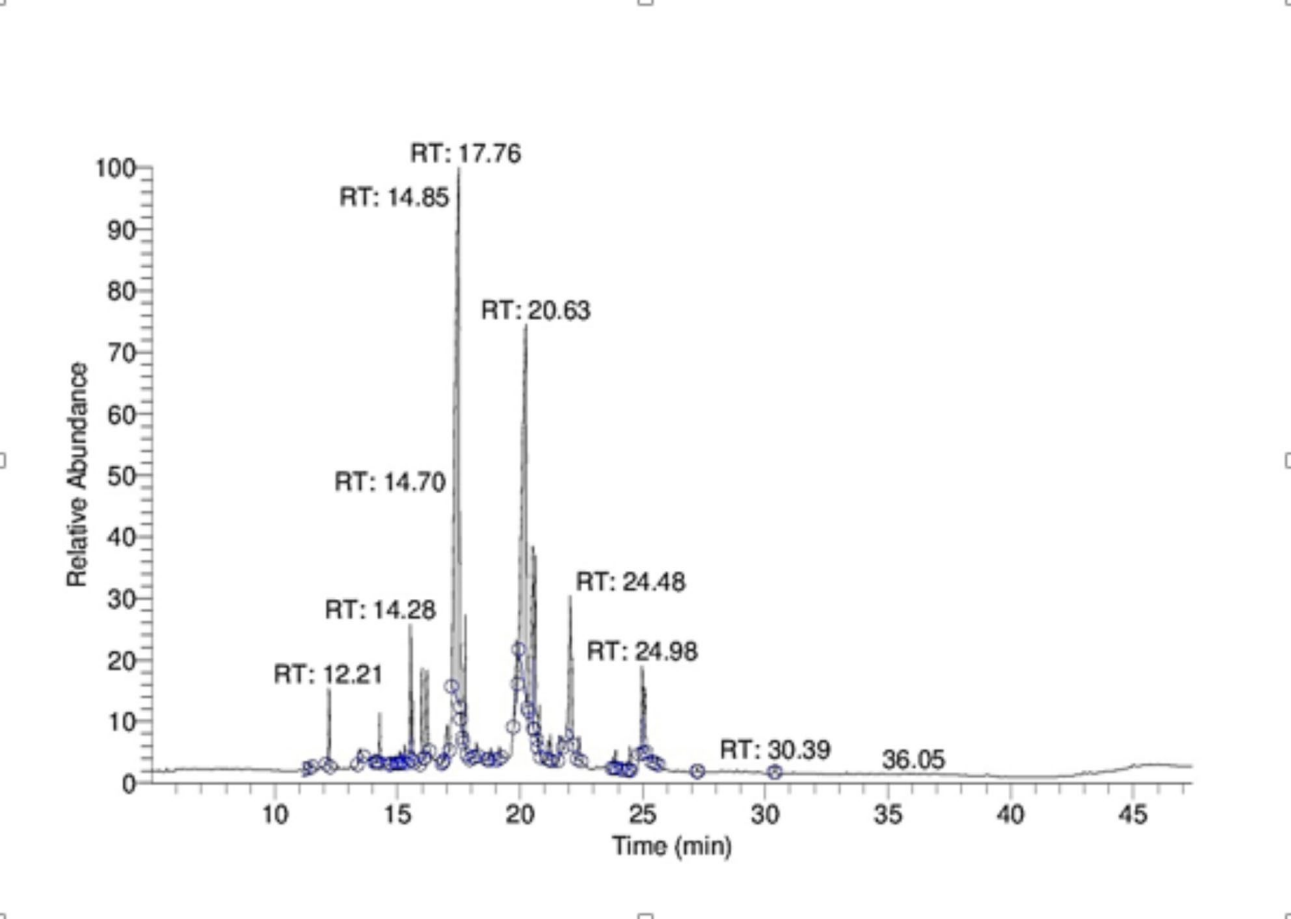
GC-MS chromatogram of derivative hydrolysate of *C. vulgaris* intracellular polysaccharides

GC-MS analysis, (Fig. 9), revealed a complex carbohydrate profile, composed mainly of neutral sugars, deoxy sugars, and minor sugar alcohols. Notable compounds and their respective retention time and area percentage are given in Table 5. A total of thirteen peaks are identified within the retention time range 7.30–25.08 min., with varying peak intensities, reflecting differences in relative abundances. The chromatogram was dominated by glucose (31.94%, RT 17.50 min.), suggesting that glucose is the major building block of the polysaccharide fraction. Other hexose sugars were also detected, including Mannose/galactose (21.63% at RT 20.23 min.) and fructose (2.41% at RT 17.76 min). The analysis revealed that pentoses like xylose (1.7-6%), arabinose (1.99%), and ribose (2.35%), indicating that the polysaccharide contains heteropolysaccharide chains in its structure. Dioxy-sugars, including rhamnose (5.222%) and fucose (3.74%), were present, which are often associated with branched polysaccharide chains and can influence biological activity. Uroic acid (3.45% at retention time of 21.68 min) was identified, verifying the existence of acidic polysaccharide groups. In addition to monosaccharides, disaccharide derivatives such as maltose/lactose-type structures were observed at later retention times (24.93–25.08 min), indicating incomplete hydrolysis of naturally occurring oligosaccharide residues. Minor peaks corresponding to glycerol/sugar alcohols (1.361.56%) were also identified. Overall, the GC-MS profile indicated that *C. vulgaris* polysaccharides are heteropolysaccharides, primarily composed of glucose, mannose/galactose, rhamnose, and uronic acids, along with smaller contributions from pentoses, deoxysugars, and disaccharide derivatives. This heterogeneous sugar composition highlights the structural complexity of CVPS, which may contribute to their reported antioxidant and anticancer activities. The same was found in Yuan, Q. et al.’s study [55].

**Table 5:** Top 13 peaks in *C. vulgaris* polysaccharide extract

Rank	RT (min)	% Area	Compound
1	17.5	31.94	Glucose (TMS derivative)
2	20.23	21.63	Mannose/Galactose
3	20.52	6	Xylose/Arabinose
4	22.04	5.22	Rhamnose
5	20.63	3.74	Fucose
6	21.68	3.45	galacturonic acid
7	17.76	2.41	Fructose
8	15.53	2.35	Ribose
9	24.98	2.21	maltose
10	16.2	1.99	Arabinose
11	16	1.77	Xylose
12	7.3	1.56	Glycerol
13	19.86	1.36	mannitol

### Biomedical application of biosynthesis PtNPs

#### CV-PtNPs as anti-coagulant agent

**Fig. 10:**
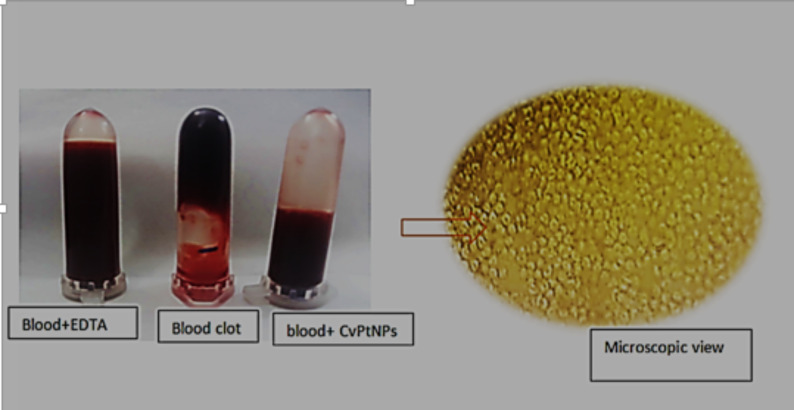
Anticoagulant activity of CV-PtNPs

The anti-coagulant efficacy of CV-PtNPs synthesized using *C. vulgaris* was assessed in comparison with EDTA and untreated controls (Fig. 10). as expected, the untreated blood sample rapidly formed a visible clot, confirming the normal physiological coagulation process. In contrast, the EDTA-treated sample completely inhibited clot formation, serving as a positive control. Interestingly, blood incubated with CV-PtNPs indicated a strong anticoagulant effect, as no clot formation was observed and the blood remained fluid closely resembling the EDTA group. Microscopic observation further supported these findings, revealing well-dispersed red blood cells without aggregation or fibrin network formation. Prior research has demonstrated that bimetallic nanostructures containing platinum and cerium, referred as PtCe NRs, possess antiplatelet properties, which inhibit blood coagulation [56].

The observed anticoagulant activity of CV-PtNPs may be associated with the intrinsic nanozyme-like properties of PtNPs, although further mechanistic investigation is required. Potential mechanisms may include interference with thrombin generation or fibrin polymerization, although these processes were not directly examined in the present study. The obtained results suggest that the biosynthesized CV-PtNPs exhibit in vitro anticoagulant effects and warrant further investigation for potential biomedical applications.

### In vitro cytotoxic study

**Fig. 11:**
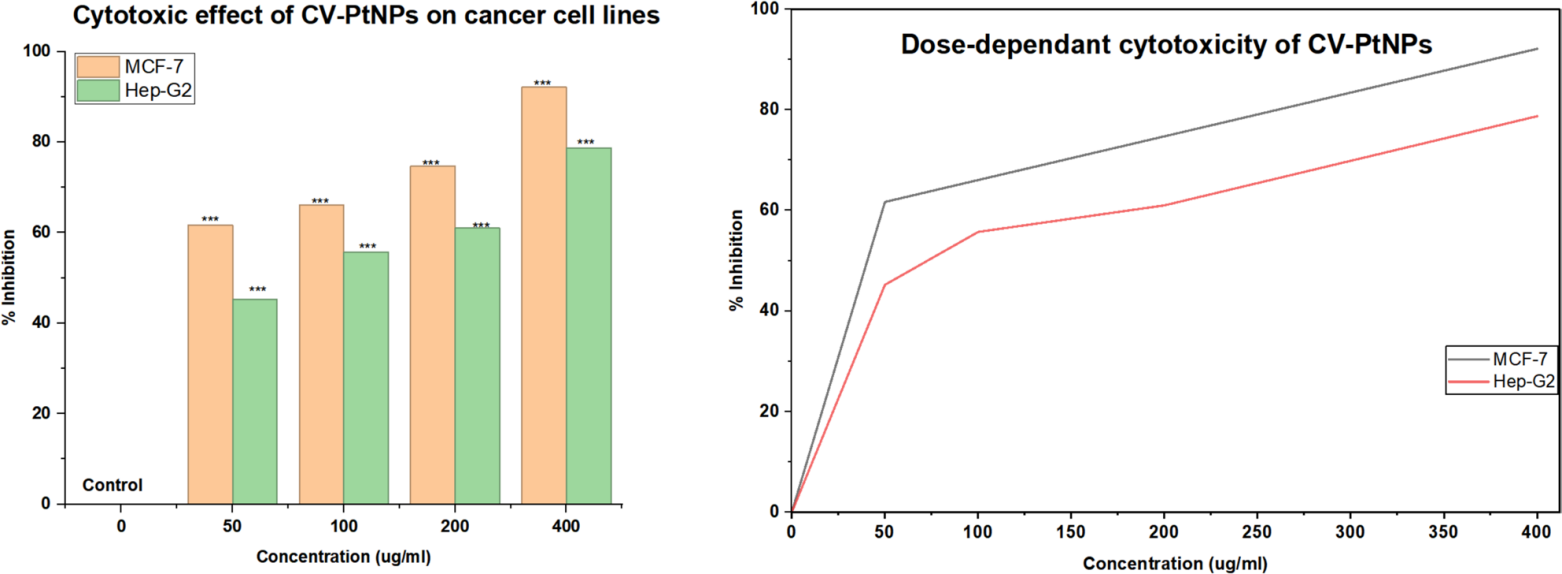
Cytotoxicity of CV-PtNPs. **A** Effect of CV-PtNPs on viability of MCF-7 & HepG-2 cell lines. Cells were seeded 37℃ for 24 h prior to treatment with CV-PtNPs Cells were either treated with (50, 100, 200, 400 µg/mL) of CV-PtNPs for 24 h or treated with 5-fluorouracil as control. Cell viability was measured through MTT Assay. **B** dose-dependant curve of CV-PtNPs against MCF-7 and HepG-2 cancer cell lines. Data are expressed as mean ± SD(*n* = 3). Statistical analysis was performed using one-way ANOVA. Asterisks indicate significant differences compared with the control : *P < 0.05, **P < 0.01, ***P < 0.001

To assess how the biosynthesized CV-PtNPs affect the viability of MCF-7 and HepG-2 cells, we performed an MTT Assay on both treated and control samples. We measured the absorbance at 490 nm. As the concentration of PtNPs increased to 400 µg/mL (Fig. 11), the viability of MCF-7 cells dropped in a dose-dependent manner, reaching 7.9%. Similarly, HepG-2 had a viability decrease to 22% at a concentration of 400 µg/mL, we determined the IC_50_ values for MCF-7 and HepG-2 are 20.9 µg/mL and 74.1 µg/mL, respectively. Cytotoxicity analysis revealed a significant, dose-dependant decrease in cell viability for all tested concentrations compared with the untreated control (*P* < 0.001). One-way ANOVA confirmed that each concentration exhibited statistically significant differences relative to the control group. These results indicate cytotoxic effects of biosynthesized CV-PtNPs against MCF-7 and HepG-2 cells in vitro. In a related study, Mohammadi et al. [57] examined chemically prepared PtNPs and observed IC_50_ values after 24 h of 6.8294 mg/mL for MCF-7 and 2.9044 mg/mL for HepG-2 cells, indicating a marked variation in their growth inhibitory effects [57].

## Limitation of the study

Despite the promising findings, several limitations of the present study should be acknowledged. First, all biological evaluations were exclusively conducted under in vitro conditions; therefore, the therapeutic efficacy and safety of the biosynthesized CV-PtNPs in living organisms remain unverified. Second, the molecular mechanisms underlying the observed anticancer and anticoagulant activities were not experimentally investigated and thus remain speculative. Third, pharmacokinetic behavior, biodistribution and potential immunological effects of the nanoparticles were not evaluated. Finally, although physicochemical characterization confirmed successful nanoparticle formation, large scale production feasibility was not assessed. Future studies should therefore focus on detailed mechanistic investigations, comparative biocompatibility studies, in vivo efficacy and safety assessments and optimization for scalable biomedical applications.

## Conclusions

This study reports the successful green synthesis and statistical optimization of platinum nanoparticles (CV-PtNPs) using intracellular polysaccharides from *C. vulgaris* as sustainable reducing and stabilizing agents. Characterization confirmed well-dispersed, quasi-spherical nanoparticles with dose-dependent anticancer activity against MCF-7 and HepG-2 cells (IC_50_ = 20.9 and 74.1 µg/mL, respectively), showing lower IC_50_ values compared with previously reported chemically synthesized PtNPs under different experimental settings. CV-PtNPs also exhibited anticoagulant activity in vitro, suggesting potential for further exploration as a multifunctional nanomedicine platform. Nevertheless, the present study is limited to in vitro evaluations and a further in vivo studies are required to fully elucidate the therapeutic potential and safety profile of CV-PtNPs.

## Data Availability

No datasets were generated or analysed during the current study.

## Abbreviations

CV-PtNPs: *C. vulgaris*-biosynthesized platinum nanoparticles
FTIR: Fourier Transform Infrared spectroscopy
TEM: Transmission Electron Microscopy
SEM: Scanning Electron Microscopy
UV-Vis: Ultraviolet-visible spectroscopy
SPR: Surface Plasmon resonance
PBD: Plackett-Burman design
ANOVA: Analysis of variance
IC_50_: half-maximal inhibitory concentration
MCF-7: Human Breast Adenocarcinoma
HepG-2: Human Hepatocellular Carcinoma
